# A Preliminary Assessment of Compassion Fatigue in Chimpanzee Caregivers

**DOI:** 10.3390/ani12243506

**Published:** 2022-12-12

**Authors:** Mary Lee Jensvold

**Affiliations:** 1 Friends of Washoe, Ellensburg, WA 98926, USA; jensvold@faunafoundation.org; 2 Fauna Foundation, Carignan, QC J3L 7M1, Canada; 3 Primate Behavior and Ecology Program, Department of Anthropology and Museum Studies, Central Washington University, Ellensburg, WA 98926, USA

**Keywords:** ProQOL, compassion fatigue, chimpanzee caregiver, husbandry, compassion satisfaction, secondary traumatic stress, burnout, animal worker

## Abstract

**Simple Summary:**

Compassion fatigue is when those in helping professions experience burnout and secondary traumatic stress in excess of the compassion satisfaction derived in interactions inherent to their occupation. It appears in medical professions and animal care workers and other occupations. This study was a preliminary assessment of the prevalence of compassion fatigue in chimpanzee caregivers using the Professional Quality of Life Scale (ProQOL-V) survey. Levels of compassion satisfaction were high but levels of burnout and secondary traumatic stress were elevated indicating potential problems in this workforce. Compassion fatigue is associated with intention to leave the profession, poor mental health, and has a negative effect on the individuals receiving care. This article suggests ways to maintain compassion satisfaction and mitigate burnout and secondary traumatic stress.

**Abstract:**

Compassion fatigue is defined as “traumatization of helpers through their efforts at helping others”. It has negative effects on clinicians including reduced satisfaction with work, fatigue, irritability, dread of going to work, and lack of joy in life. It is correlated with patients’ decreased satisfaction with care. Compassion fatigue occurs in a variety of helping professions including educators, social workers, mental health clinicians, and it also appears in nonhuman animal care workers. This study surveyed caregivers of chimpanzees using the ProQOL-V to assess the prevalence of compassion fatigue among this group. Compassion satisfaction is higher than many other types of animal care workers. Conversely, this group shows moderate levels of burnout and secondary traumatic stress; higher levels than other types of animal care workers and many medical professions. While compassion fatigue has an effect on the caregiver’s experience, it has potential to affect animal welfare. Caregivers are an integral part of the chimpanzee social network. Compassion fatigue affects the caregiver’s attitude, this could in turn affect the relationship and degrade the experience of care for captive chimpanzees. Compassion fatigue can be mitigated with professional development, mindfulness training, interrelationships among staff, and specialized training. This preliminary assessment indicates the work ahead is educating caregivers about compassion fatigue and implementing procedures in sanctuaries to mitigate burnout and secondary traumatic stress.

## 1. Introduction

Professional Quality of Life (ProQOL) is the experience of workers in helping professions. It is comprised of both compassion satisfaction (CS) and compassion fatigue [1]. CS “is characterized by feeling satisfied by one’s job and from the helping itself. It is characterized by people feeling invigorated by work that they like to do… They experience happy thoughts, feel successful, are happy with the work they do, want to continue to do it, and believe they can make a difference [1] (p. 21)”.

In contrast compassion fatigue (CF) is the “traumatization of helpers through their efforts at helping others” [2] (p. E56) and is the negative aspect of caring occupations. It is comprised of two factors, Burnout (BO) and Secondary Traumatic Stress (STS). BO involves “feelings of unhappiness, disconnectedness, and insensitivity to the work environment. It can include exhaustion, feelings of being overwhelmed, bogged down, being ‘out-of-touch’ with the person he or she wants to be, while having no sustaining beliefs.” [1] (p. 21). It is caused by exposure to prolonged high levels of work stressors, life demands, and poor work life balance [2]. STS is when caregivers are “preoccupied with thoughts of people one has helped. Caregivers report feeling trapped, on edge, exhausted, overwhelmed, and infected by others’ trauma. Characteristics include an inability to sleep, sometimes forgetting important things, and an inability to separate one’s private life and his or her life as a helper—and experiencing the trauma of someone one helped, even to the extent of avoiding activities to avoid reminders of the trauma [1] (p. 21)”. STS is caused by exposure to trauma or suffering of others or direct trauma [1]. 

Moderate and high levels of BO and STS create CF. Overall, CF has negative effects on care workers including reduced satisfaction with work, fatigue, insomnia, irritability, dread of going to work, depression, sadness, grief, isolation guilt, relationship conflicts, low motivation, feeling empty, numb, anxiety, poor focus, cynicism, suicidal thoughts, work tardiness and absenteeism, and lack of joy in life [3,4]. While these symptoms appear in CF and BO, CF is more than simply burnout. The key difference is that the occupations in which CF appears involve interactions; there is a relationship between the caregiver and care receiver [2]. CF occurs in individuals with occupations that involve caring for individuals that have experienced trauma or extreme suffering and spans a variety of helping professions including educators [5], social workers [6], mental health therapists [7], physicians, nurses including ICU and oncology [8], and emergency room workers [9]. These are occupations that involve interactions with and care for individuals who potentially have experienced trauma. CF also appears in nonhuman animal care workers, including shelter workers, laboratory workers, and veterinarian practitioners [3,10,11,12]. These environments involve trauma such as euthanasia or abandonment. 

People in caring occupations often generate satisfaction from their work and it is these very interactions that afford that satisfaction [2]. High levels of CS can offset high levels of CF, to an extent. Thus, it is important to consider the levels of all three aspects, CS, BO, and STS in discussion of CF and ProQOL [1].

CF can affect work performance. Clinical mental health workers who experience CF may be more likely to make misdiagnoses, plan treatment poorly, or abuse clients compared to those who are not experiencing it [13]. Providers may show lack of empathy [14]. CF also is reflected in the experience of the patient. Hospital patients reported less satisfaction with health care on units where nurses had higher levels of BO. Conversely the patients reported higher satisfaction on units where nurses had lower levels of BO [15,16,17]. As stated, BO is one component of CF.

CF has long been a concern for nonhuman animal care workers. It is prevalent and concerning in veterinarians who perform euthanasia and witness cruelty [10,12,18,19]. It has extended to other kinds of animal care workers including nonhuman animals care workers shelter workers, wildlife rehabilitators, laboratory workers, and primate caregivers [19,20,21,22,23]. To date only one published article has evaluated CF in non-human primate (NHP) caregivers. Schlanser and colleagues [24] surveyed 67 Army laboratory animal care specialists working with a variety of species. Those working with (NHP) had significantly higher levels of CF than those who worked with other species. The present study is the first to evaluate the prevalence of CF specifically in chimpanzee caregivers. At sanctuaries and a few laboratories, chimpanzees are the only species at the facility, as contrasted for example at zoos which house many different species. At these sanctuaries and laboratories, caregiver only care for one species as contrasted to zoos where they often care for multiple species. Thus, we have caregivers exclusively working with chimpanzees which may present different challenges for ProQOL than other types of animal care workers. 

There are approximately 1327 chimpanzees in North America. Of those, 921 live in sanctuaries accredited by the Global Federation of Animal Sanctuaries (GFAS) and zoos accredited by the American Zoological Association (AZA) [25,26]. An additional approximate 406 live in laboratories, and unaccredited facilities [25]. This study surveyed caregivers of chimpanzees to measure ProQOL. The hypothesis was that CF would be roughly the same in chimpanzee caregivers as in other animal care workers. 

A standard measure of CF is the Professional Quality of Life Scale (ProQOL-V) [1,6,27]. This 30 item self-report questionnaire assesses CS, BO, and STS which creates an overall profile of ProQOL. There are several versions and the latest one is the 5th, ProQOL-V. The survey was developed by Stamm and is now owned by the Center for Victims of Torture [1]. It is free to use. It has good construct validity in hundreds of studies [1] and since it appears in many studies [27], it allows for comparison between occupations.

## 2. Materials and Methods

Participants were recruited through online dissemination on social media with a link to the survey. Additionally, the researcher shared an email announcement to professional acquaintances who have cared for chimpanzees primarily in the sanctuary setting. The email asked them to share the survey link with others and on social media to create a snowball effect. This method of recruitment was similar to that of other studies that assessed ProQOL in animal care workers [10,19,21,22,23]. It was designed to maintain anonymity and ensure participants did not feel coerced by employers. Participation was voluntary and there was no compensation for participation. Respondents were required to be 18 years or older and currently caring for chimpanzees. There was no stipulation for the workplace setting, location, etc.

The survey was administered online through Qualtrics software (February, 2022) (Qualtrics XM Software Company, Provo, UT, USA) which insured responses were anonymous. This methodology, including the details of recruitment and survey administration, was approved through the Human Subject Review Committee at Central Washington University in Ellensburg, WA, USA. That committee requires that surveys which are anonymous utilize Qualtrics. Online surveys were used in several other studies of CF in animal care workers [10,19,21,22]. Participants provided informed consent to initiate the survey. In the informed consent participants attested that they were 18 years of age or older and currently working with captive chimpanzees. The survey concluded with a debriefing that contained brief information about CF.

The survey was 31 questions. The first question was demographic and the subsequent 30 questions were the survey items on the ProQOL-V. The first question on the survey asked the number of years the respondent had worked with chimpanzees. The ProQOL-V contains 3 subscales, CS, BO, and STS. Each question assessed one of the subscales. For example, a question from the CS subscale asked, “I get satisfaction from being able to care for chimpanzees”. A question from the BO subscale asked, “I feel trapped by my job as a caregiver”. A question from the STS subscale asked “I jump or am startled by unexpected sounds”. Responses to ProQOL-V were indicated by a numerical response on a Likert scale. Respondents indicated 1 for ”never” or 5 for “very often”. Instructions stated to rate the prevalence of each questioned item in the last 30 days. This produced a numerical score for each question. See Appendix A for a link to the ProQOL-V survey.

The researcher obtained permission from the ProQOL office at to use the survey and to change the word “people” to “chimpanzees” and “helper” to “caregiver”. The survey was open for participants from 27 February 2022 to 12 April 2022.

The ProQOL-V survey scores were calculated as per the instructions in Stamm [1]. The process generated a separate score for each subscale for each individual survey. First, the scores of 5 questions (survey items 1, 4, 15, 17 and 29) were reversed. A score of 1 was reversed to 5, 2 to 4, 4 to 2, 5 to 1, and 3 remained a 3. Next the individual Likert scores for each subscale were summed including the reversed (rather than original) scores. The subscale for CS was survey items numbered 3, 6, 12, 16, 18, 20, 22, 24, 27, and 30. The subscale for BO was survey items numbered 1, 4, 8, 10, 15, 17, 19, 21, 26, and 29. The subscale for STS was survey items numbered 2, 5, 7, 9, 11, 13, 14, 23, 25, and 28. This produced a total score for each subscale. That was the raw score.

Stamm [1] describes several descriptive statistics to present survey results. There are continuous measures which are t-scores and raw scores. There also are categorical measures which Stamm refers to as “cuts”. The categories are based on the 25th and 75th percentiles [1]. Raw scores below 23 were categorized as low, 23–41 as medium, and 42 and above as high. Stamm recommends use of continuous scores. This researcher calculated each type of descriptive statistic, t-scores, raw scores, and cuts to present data that were comparable to as many other studies that used the ProQOL-V as possible. The researcher calculated the mean and standard deviation (SD) of the raw scores for each subscale. The researcher converted raw scores into t-scores for each subscale. The researcher calculated the mean and SD of the t-scores for each subscale. The researcher calculated Pearson r correlations on the number of years of experience and the raw score for each subscale. The researcher used Excel v16 for all calculations. All of the completed survey data were included in the analysis. There were no outliers.

Stamm [1] provides guidelines for interpretation of scores. CS scores above 23 indicate a high level of professional satisfaction. A score below that indicates the individual is not deriving satisfaction from their job. A BO score below 23 indicates positive feelings about effectiveness at work. A score above 41 indicates a high level of BO. STS scores above 43 indicate a high level of secondary trauma or fear in the workplace.

Each subscale measures a separate construct and has high construct validity. There is some shared variance between BO and STS (34.0%) but STS is distinct in measuring fear [1].

## 3. Results

There were 61 completed surveys. The average number of years respondents worked with chimpanzees was 5.57 and ranged from 1–25. There were 39 with 1–5 years, 18 with 6–15 years, and 4 with more than 15 years. Table 1 shows the descriptive statistics for each subscale.

Table 2 shows the number of raw scores in the categories of low, medium, and high for each subscale. It also shows the percentage for each subscale. For CS no respondents scored low and 63.9% scored in the medium category. For BO no respondents scored in the high category and 77.1 % scored in the medium category. For STS 3.27% of respondents scored in the high category and 68.9% scored in the medium category.

Pearson product correlations compared the number of years of experience working with chimpanzees with each subscale score. The relationship between years of experience and CS was not significant r = −0.097, *p* = 0.45. The relationship between years of experience and BO was not significant r = 0.0855, *p* = 0.51. The relationship between years of experience and STS was not significant r = −0.1294, *p* = 0.321.

## 4. Discussion

### 4.1. Comparison with Other Occupations

The next subsections will discuss the meaning of the scores for each subscale in the ProQOL-V. It will compare the scores of chimpanzee caregivers to scores of other kinds of animal care workers. Human medical care requires adherence to safety protocols while delivering care to vulnerable individuals. Human medical care and chimpanzee care have parallels in general objectives, an interactional setting, and potential for a dynamic and stressful workplace. CF is well studied in medicine [2] so these studies provide a way to compare the prevalence of CF in chimpanzee caregivers to other occupations. The means and distribution of the categories will be used for comparison.

#### 4.1.1. Compassion Satisfaction

**Figure 1:**
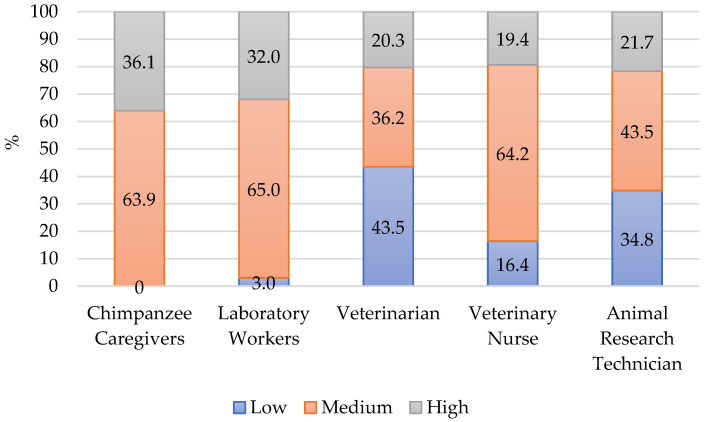
Percent of respondents in each category for CS. Laboratory Workers (N = 170) [21], Veterinarian (N = 69) [12], Veterinary Nurse (N = 23) [12], Animal Research Technician (N = 23) [12].

Over 1/3 of chimpanzee caregivers showed high levels of CS and none had low levels. This means that overall they are deriving satisfaction from their occupation in chimpanzee care. Figure 1 shows the percentage of scores in the categories of high, medium, and low in various animal caregiving occupations including chimpanzee caregivers from this study. Thurston [21] surveyed 170 animal laboratory workers during the COVID-19 pandemic, which had impacts on the animal care community. This online survey included veterinarians, veterinary nurses, and husbandry technicians but did not indicate which species. This study recruited participants through direct emails and list serves. It was chosen for comparison because of the impacts of COVID-19 also may have affected chimpanzee caregivers at the time of data collection in this study. Despite the additional stress of the pandemic [21,28] many of these laboratory workers had high levels of CS. Scotney and colleagues [12] surveyed 229 animal care workers from a variety of occupations such as veterinarian practices, research technicians, and shelter workers. They recruited participants at animal related conferences and handed them the survey. The authors presented the data by occupation which provided comparable data to this study. Veterinarians [12] in that study had the largest percent of low scores and few scored high in CS.

These studies contrast to Schlanser and colleagues [24] who surveyed 65 Army corps veterinarians and animal care specialists working in Department of Defense Army animal research laboratories. Recruitment was through emails sent to active-duty Army veterinarian personnel and to closed Facebook groups. Participants completed the survey online. Anyone with the link could complete the survey. Among the specialists 52% scored high levels of CS and none scored low levels. In this survey respondents may have worked with laboratory animals anytime in the previous 5 years. This is in contrast to the current study and others in which the participants were currently working in animal care. This may have contributed to the high level of CS in the Army workers.

The mean CS score for chimpanzee caregivers was 39.26 (Table 1). This was less than a survey of NHP sanctuary workers (42.0, N = 46) [20]. It was equal to animal care workers (39.2, N = 229) [12], and higher than veterinarian professionals (37.8, N = 136) [19], and all of the laboratory workers (38.0, N = 170) [21]. This shows the same pattern as the comparison of categories. Compared to other animal related occupations, the data in this study indicates that chimpanzee caregiving is a highly satisfying occupation.

Cavanagh and colleagues [29] presented a comprehensive review of studies of CF in human medical professions. In the 28 studies that used the ProQOL-V the pooled mean CS score in the meta-analysis was 41.8. Within that population, variation occurred among the different medical specialties. Of note were physical therapists (40.0) [30]; transplant nurses (40.0) [31]; registered nurses (37.0) [32]; and resident physicians (40.0) [33]. The lowest scores in this review were among palliative care nurses (35.2) [34] (see [29] Table 1 for review). In comparison to human medical care, chimpanzee care is highly satisfying.

CS is derived from the interaction between carer and receiver. Chimpanzees and human share a long evolutionary history and non-verbal behaviors such as facial expressions and postures [35]. This may facilitate the ability to communicate as compared to other species in animal care. Additionally, the relationships between caregivers and chimpanzees are lasting. This is in contrast to medicine in which different patients come through. Perhaps this is another quality of chimpanzee care that makes it so highly satisfying.

#### 4.1.2. Burnout

**Figure 2:**
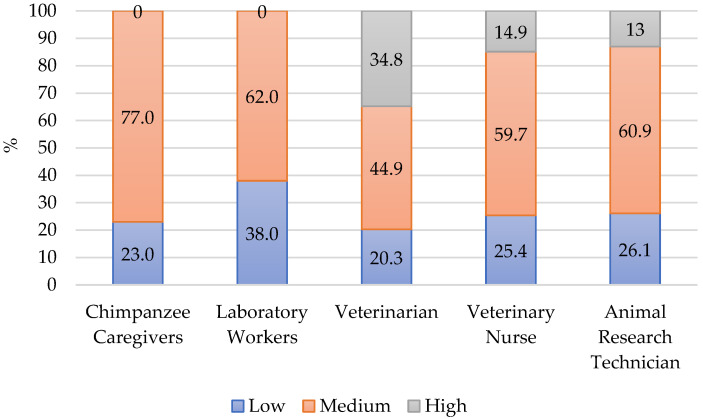
Percent of respondents in each category for BO. Laboratory Workers (N = 170) [21], Veterinarian (N = 69) [12], Veterinary Nurse (N = 23) [12], Animal Research Technician (N = 23) [12].

The percentage distribution of BO scores into the high, medium, and low categories appears in Figure 2. The highest proportion (77%) of chimpanzee caregivers scored medium on BO and no respondents scored high. While veterinarians, veterinary nurses, and animal research technicians [12] had a greater percentage of workers scoring high BO, if considering a combination of medium and high, chimpanzee caregivers were like veterinarians. The overall profile of CF in veterinarians presents high risk for burnout as the high BO is coupled with low CS. Veterinarians are four times more likely than the general population to commit suicide and this high risk has been a concern for this occupation [36].

The mean BO score for chimpanzee caregivers was 27.46. This was equal to overall veterinary workers (27.6) [19] and higher than NHP sanctuary workers (24.0) [20], laboratory workers during the COVID-19 pandemic (25.0) [21], and much higher than US Army laboratory workers (20.8) [24]. Although, as noted earlier, within the Army corps, those who worked with nonhuman primates scored significantly higher on BO than those who worked with other taxonomic groups.

In Cavanagh and colleagues’ review, the pooled ProQOL-V mean BO score for medical workers was 28.4 [29]. There was wide fluctuation between different medical specialties with some groups having very high scores such as surgeons (25.3) [37] and palliative care nurses (27.3) [34]. Lower scores occurred in general physicians (19.4) and nurses (20.6) [38], and oncology nurses (22.5) [1]. In this review [29] there were 10 studies above the pooled mean and the greatest variability between studies was in BO as compared to CS and STS. All the means in medical professions were lower than chimpanzee caregivers. Again, we see evidence that BO is high for chimpanzee caregivers.

#### 4.1.3. Secondary Traumatic Stress

**Figure 3:**
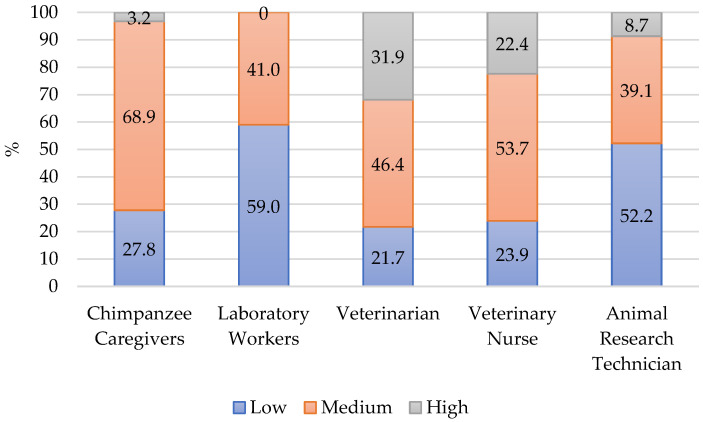
Percent of respondents in each category for STS. Laboratory Workers (N = 170) [21], Veterinarian (N = 69) [12], Veterinary Nurse (N = 23) [12], Animal Research Technician (N = 23) [12].

The percent distribution of STS scores into high, medium, and low categories appears in Figure 3. The highest proportion (68.9%) of chimpanzee caregiver participants scored medium on STS and 3% scored high. The percentage of combined medium and high scores was greater than laboratory workers [21] and animal research technicians [12]. The profile for chimpanzee caregivers overall was similar to veterinarians and nurses in that there were about 25% with scores in low cut. At the other extreme, chimpanzee caregivers did have fewer high scores than veterinarians and nurses. This may be a result of regular encounters with euthanasia and death. Chimpanzee caregivers do encounter death but it is not a regular daily occurrence as in veterinary medicine.

The mean score for STS for chimpanzee caregivers was 27.56 which was like veterinary professionals (27.0) [19]. The mean was higher than other NHP sanctuary workers (24.0) [20], Army laboratory workers (18.9) [24], laboratory workers during the COVID-19 pandemic (21.0) [21], and overall general animal care workers (24.6) [12]. Like in the BO scores, Army laboratory workers who worked with NHP scored higher in STS than those who worked with other taxonomic groups [24].

In Cavanagh and colleagues’ review, the pooled mean score on STS for medical workers was 25.8 [29] which also was lower than the mean score for chimpanzee caregivers. Palliative care nurses (26.1) [29] were the only group in the review that had a mean score approaching that of chimpanzee caregivers. Like the comparison for BO, the level of STS raises concern about the overall ProQOL of chimpanzee caregivers. It indicates some exposure to trauma or secondary trauma which is discussed later in the article.

#### 4.1.4. The Interaction of Subscales

The developers of the ProQOL indicate the test is not a diagnostic tool [1] instead it is a screener to indicate potential problems. In assessing ProQOL, researchers evaluate each of the scales independently. At the same time ProQOL [1] is a blend of factors so the scales are considered in relationship to each other. High levels of CS can offset high or medium levels of BO and STS and those two combined are CF. For chimpanzee caregivers there was a large percent of individuals with medium STS and BO scores, which is mitigated somewhat with the large percent of high CS scores. This high CS is something to be maintained and promoted. The data from this survey indicates that chimpanzee caregivers would benefit from support for BO and STS. Organizational policies and practices, such as promoting a work-life balance, can reduce BO [15] while at the same time organizational factors such as job demands, workload, haste and complexity of work, and work-life conflicts can contribute to it [19,39]. Managers and leaders can change many of these practices. While much of the work of improving ProQOL is up to the individual, there are easy ways to provide outside support. BO is a concern in nursing and human health care occupations and is well studied [8,29]. It can provide a model for captive chimpanzee staff management and approaches to care which will be discussed later in this article.

### 4.2. Years of Experience

Scotney and colleagues [12] found a significant effect of years of experience and levels of CF. For CS, animal care workers with 6–15 years of experience had more scores in the average and low category than workers with more or less experience. Animal care workers with 6–15 years of experience had significantly higher scores on BO than those with more or less experience. There was no effect for STS. The data in this study with chimpanzee caregivers found no relationship on any of the scales. Army laboratory workers [24] also showed no relationship between years of experience and CF. Stamm [1] found no correlation between years in occupation and ProQOL in the general public. The representation of years of experience among the participants in this study was uneven which may have affected the results. This is an area that deserves future research. The effect of the type of training and education also would provide an interesting area for future studies.

### 4.3. Compassion Fatigue Impacts on Quality of Care

While CF has an effect on the individual caregiver’s experience, it also has potential to affect animal welfare [40]. The patient’s perceived quality of care can inform us about the effects of high CF on healthcare providers. A high level of BO in nurses in 40 hospital units was directly related to patients’ reported satisfaction of care [15]. Zajac and colleagues [17] provided an intervention of grief support for oncology nurses. Surviving patient satisfaction was higher on nurse units that had received the intervention versus those units that had not received it. These researchers indicate that addressing BO will improve patient satisfaction. Additionally, it improves staff retention as often individuals who experience high levels of BO indicate an intention to leave the job [19]. At the time of this writing, sanctuaries and zoos are experiencing chimpanzee caregiver shortages [41]. The data indicates moderate levels of BO in this same community. Addressing BO on an organizational level might also help with staff retention and mitigate shortages.

Wei and colleagues [42] interviewed parents of pediatric heart patients on their care. They found that the parents’ perceptions of quality care were related to perceived “competence”, “altruism”, “responsibility”, and “empathy”. These are valuable professional competencies. “Competence” is that caregivers know what they are doing; they are skilled. This is generated from being well trained and experienced. “Altruism” is treating families with dignity and respect. Families felt that they were treated as individuals, not just as a number. ”Responsibility” is not passing the buck. In this, caregivers are empowered to solve the problem and ensure that action is taken even if they are not the person who is the actual fixer. For example, the caregiver follows up to make sure the plumber has come to fix the drain. “Empathy” for the parents included efficiency which circles back to “competency”. It also included an understanding of what the patient was experiencing. In terms of caring for chimpanzees, this is an acknowledgment of the chimpanzee’s experience. Chimpanzees understand human speech [43,44] so speech and nonverbal behaviors can be used to communicate. Caregivers can specifically acknowledge chimpanzees’ behaviors, such as waiting a turn for meal service. Also, voice quality and language can indicate perceptions of interpersonal relationships.

Finally, Wei and colleagues [42] addressed the issue of power to powerlessness, which is a component of medicine as well as captive chimpanzee care. No matter how we view it, chimpanzee caregivers hold the keys and chimpanzees are captive, which is an imbalance of power [45,46]. Environmental enrichment programs are a way to boost control as is outdoor access [47]. Additionally, chimpanzees may feel empowered when caregivers integrate choices into interactions and care.

Perry [48] conducted interviews with exemplary oncology nurses, who were nurses who received high ratings from fellow nurses. The exemplary nurses also had scored low for CF. One theme from the interviews was moments of connection between nurse and patient. These connections bolstered CS in the nurses and decreased CF. Nurses reported recognizing similarities between themselves and patients, and that helped them understand how the patient wanted to be treated. Caregivers’ recognition of the status and intelligence of chimpanzees can perhaps increase “empathy” and connections while also bolstering CS in the caregiver.

Like for nurses [4,13,14,48], CF can affect the caregiver’s attitude [3] which could in turn affect the relationship and degrade the chimpanzees’ experience of care. Caregivers are an integral part of the chimpanzee social network [49,50] and these relationships are a way to foster CS, connections, and quality of care. One aspect of these relationships is the caregiver’s understanding of chimpanzee behaviors and their meaning and context [51] as well as physiology. This allows caregivers to understand the chimpanzees’ behavioral context of the interaction, for example playful versus aggressive, and monitor arousal levels. This also allows caregivers to better understand what chimpanzees are communicating and their needs which will lead to improved service and care. Finally, it allows caregivers to better understand relationships between chimpanzees. For example, understanding that grooming indicates positive relationships and pant grunts are clues to understanding hierarchy among the chimpanzees.

Caregivers can incorporate chimpanzee behaviors into their own interactions with chimpanzees [52,53]. For example, food grunting to generate enthusiasm about a tasty meal. Pant hoots to generate excitement about an enrichment activity. Grooming mouth sounds to increase intensity in that interaction. Laughter to create a playful interaction. Conversely submissive behaviors particularly in greeting and in high arousal situations, can help to de-escalate tension.

Relationships between caregivers and animals are affected by interactions with caregivers. Pigs [54], cows [55], rhesus monkeys [56], and chimpanzees [57,58] all showed positive responses to interactions with caregivers. Animal care workers whose job responsibilities included basic caregiving tasks such as cleaning and serving meals had high levels of CS as compared to veterinarians and veterinary nurses. These activities are the ones that involve the most interaction with the animal patient [12]. This points to the importance of the relationship to human well-being as well. The relationships are a way to foster connection and increase CS. Additionally, these relationships can improve caregivers’ attitudes towards the animals for which they care [59].

### 4.4. Trauma

Trauma includes obvious things like work in war zones or with refugees, or more subtle things like work with individuals who are injured or ill, euthanasia, laboratory experimentation, or simply captivity [60]. Some captive chimpanzees manifest abnormal, stereotypical, or self-injurious behaviors [61,62,63,64,65] which are associated with trauma in humans [66,67,68]. Many captive chimpanzees have experienced some level of trauma such as separation from mother in infancy or life history events (experimentation, entertainment, neglect) [26,45]. Chimpanzee caregivers then often are exposed to individuals who have experienced trauma which is the key operative in STS. Chimpanzee caregivers may witness high levels of aggression and injury between chimpanzees or toward humans. Safe protocols are designed to mitigate injury to humans and chimpanzees, but the unintended does happen, hopefully rarely. Grief also is a source of trauma. Caregivers likely will experience grief as death is part of any life and chimpanzees in their care may die, particularly with the aging population of chimpanzees in US sanctuaries [46]. The data in this study supports that STS is part of the chimpanzee care environment. It is useful to recognize these very real aspects of chimpanzee care in addressing STS. With this knowledge caregivers and managers can move towards means to mitigate STS. For example, grief support after resident deaths.

### 4.5. Processes to Mitigate Compassion Fatigue

Caregivers and their organizational managers can take many actions to improve ProQOL. One is by increasing CS and another is by combating BO and STS. Some activities will serve both purposes. There are many resources [68] to discover these processes including books [3], websites [69,70,71,72], and videos [72].

CS can be increased with counseling, positive co-worker dynamics, direct animal care, personal care routines [1,73], and gratuity practices to name a few. A gratuity practice is an intentional focus on positive aspects of life. It increases optimism, beneficial emotions, positive affect, and prosocial behavior [74]. An example is a daily gratuity log in which an individual writes three things they are grateful for each day. Over time the practice refocuses attention towards the positive and provides relief from a focus on negative aspects of the workplace. Gratuity exercises are easily accessed online [75].

Mindfulness practices are ones that direct attention to the immediate environment [3] allowing a break from stress and worry [76]. Mindfulness can be a minute spent on focusing on the sensation of air moving over the lips, or noticing sounds in the immediate environment, or mindfulness walking in which deep attention is focused on the sensations during slow walking. These practices are associated with positive outcomes in health care professions [76]. The ProQOL website [69] provides suggestions for activities and more in-depth reading and practices are available [77].

There are many other types of practices that can reduce stress. Breathing exercises decrease heart rate, lower and stabilize blood pressure, decrease anxiety and anger [78,79,80,81], and often are part of mindfulness practices. Stretching, progressive muscle relaxation, visualization, and grounding techniques are all interventions to reduce stress [1,3,18]. Emotional Freedom Technique (EFT) is a stress relief practice based on tapping acupressure points [81]. It is effective in reducing cortisol and is used in counseling therapy as well as trauma treatment [81]. Yoga is an ancient practice that combines relaxation, breathing, physical exercise, and meditation [80,82,83]. Other suggestions to improve personal well-being include regular physical exercise, self-care routines, adequate sleep, and a well-balanced diet [3,84].

A systematic analysis of interviews with physicians indicated that healthy boundaries and work-life balance are important aspects of self-care [85]. While guarding against BO, work-life boundaries allowed restorative activities such as time with friends and time away from work to reset. Figley and Roop [18] describe this as “detachment” in that the worker has time detached from work. The ProQOL website [69] offers resources to develop skills in this regard. Some are as simple as changing out of work clothes when off duty. Sanctuary managers also can foster this for example by respecting time off, encouraging employees to take all of their vacation, or not expecting email responses on days off. Strong work-life boundaries are an important aspect of avoiding BO and improving CS [85].

For caregivers to sustain in their occupation, the approach should be one of compassion versus empathy. Dowling [86] describes the contrast between empathy and compassion in veterinary medicine. “Compassion goes beyond feeling with the other to feeling for the other.” (p. 750). Empathy stimulates pain centers while compassion stimulates reward centers and affiliative processes [87]. Empathy is important as it fosters connection, but compassion goes beyond by helping the partner resolve the pain. Animal care workers often are empathic which is being sensitive and aware of the feelings of others [18] sometimes to the point of feeling pain [86,88]. In contrast, to have compassion means they understand it, but they don’t feel the pain. Researchers [86,89,90] have suggested compassion meditation as a way to increase compassion. Some evidence lends some support to this contention. Compassionate training and meditation practice increased activation of brain regions associated with inferring emotions in others [89]. A review of the relationship of compassionate meditation on mental health showed a practice in compassionate meditation was related to improvements in affect, depression, anxiety, and anger regulation [91]. Meditation promotes prosocial behavior and alters cortical activation in regions associated with social cognition and emotional regulation [92,93]. There is evidence to support that a compassion meditation practice can increase brain activity that corresponds to aspects of empathy and, more so, compassion. At the very least it can be helpful to caregivers to understand the differences in these to concepts in their application to chimpanzee- and self-care.

### 4.6. Future Directions

There is recent recognition throughout animal care that to provide for good animal welfare, we need to ensure good welfare of their caregivers [39,40]. This study was to determine the prevalence of CF in chimpanzee caregivers and is a first step in this regard. Future research can provide improvements to this study and extend the findings. A future survey directed towards specific organizations such as zoos, sanctuaries, and laboratories would ensure equal representation across facility types. It also would likely increase the sample size, which was relatively low in this study. This study, as preliminary and to avoid risking anonymity, did not collect any demographic information. A larger study could collect more demographic data such as workplace, location, training, and education. This would offset any potential bias that may have occurred in this study, such as years of experience and response bias. It is a balance to gather detailed information and to have the survey delivered by supervisors while ensuring that participants feel their participation is voluntary and that they will answer honestly. Determining the numbers of chimpanzee caregivers in North American, Europe, Africa, and Asian is a study within itself. Thus, we do not have an idea of the relative rate of response in this study. But certainly, the number of caregivers is lower than human healthcare and veterinary care, so the overall pool is smaller. Which again is a potential concern for ensuring anonymity in acquiring demographic responses.

The large percentage of respondents with moderate levels of BO and STS in this study is a concern. A mixed methods could include interviews and open-ended questions to get more information about the sources of BO and STS. Then managers can begin the process of intervention in chimpanzee care. This study presents a first step in this direction.

## 5. Conclusions

The data in this study show that chimpanzee caregivers experience high levels of CS. This lays an excellent groundwork to improve their ProQOL. It also found a moderate level of BO and STS. The work ahead is educating caregivers about CF and ProQOL and implementing procedures in sanctuaries to mitigate STS and BO. Resources from granting agencies can be directed to this regard to develop programs and support in these areas.

## Figures and Tables

**Table 1:** Descriptive statistics for raw and t-scores.

	CS	BO	STS
Range raw	25–50	10–40	15–43
Mean raw	39.26	27.46	27.56
SD raw	6.50	6.76	6.81
Range t-scores	27.88–66.66	22.92–69.45	31.27–73.02
Mean t-score	50.0	50.0	50.0
SD t score	10.08	10.49	10.15

**Table 2:** Number of raw scores (n) and percent in each subscale.

	CS	BO	STS
	n (%)	n (%)	n (%)
Low	0	14 (23.0)	17 (27.9)
Medium	39 (63.9)	47 (77.0)	42 (68.9)
High	22 (36.1)	0	2 (3.3)

Low = 0–22; Medium = 23–41; High = 42 and higher [1].

## Data Availability

The data presented in this study are available on request from the corresponding author. The data are not publicly available due to privacy.

## Appendix A

The ProQOL-V is available at https://img1.wsimg.com/blobby/go/dfc1e1a0-a1db-4456-9391-18746725179b/downloads/ProQOL_5_English.pdf?ver=1657301051771 (accessed on 7 December 2022).
